# Neuropeptides as regulators of bone metabolism: from molecular mechanisms to traditional Chinese medicine intervention strategies

**DOI:** 10.3389/fphar.2025.1516038

**Published:** 2025-02-28

**Authors:** Qing Lin, Biyi Zhao, Jiajia Huang, Rumeng Chen, Weipeng Sun, Qianyun Ye, Li Yang, Xiaofeng Zhu, Xiaoyun Li, Ronghua Zhang

**Affiliations:** ^1^ College of Traditional Chinese Medicine, Jinan University, Guangzhou, Guangdong, China; ^2^ Guangdong Provincial Key Laboratory of Traditional Chinese Medicine Informatization, Guangzhou, Guangdong, China; ^3^ The First affiliated Hospital, Jinan University, Guangzhou, Guangdong, China; ^4^ College of Pharmacy, Jinan University, Guangzhou, Guangdong, China

**Keywords:** neuropeptide, bone formation, bone resorption, osteoporosis, traditional Chinese medicine

## Abstract

Osteoporosis (OP) is a complex bone metabolism disorder disease that affects the skeleton, nervous system, muscles, and multiple tissues. Neuropeptides, which are endogenous substances derived from both bone and brain, play a critical role in maintaining the balance of bone metabolism. This review summarizes research conducted from 1986 to 2024 on the pathological mechanisms of neuropeptides and their receptors in the context of OP. Specifically, the roles of Neuropeptide Y, Vasoactive Intestinal Peptide, Calcitonin Gene-Related Peptide, and Substance P and their receptors in key processes of OP were examined, including their function of bone formation and resorption, osteoblast differentiation, and osteoclast differentiation. Our study showed that these neuropeptides could promote bone formation and inhibit bone resorption, while their receptors in osteocytes exhibit distinct functions, indicating complex regulatory mechanisms that require further investigation. Additionally, we summarize the progress of Traditional Chinese Medicine (TCM) formulae, single TCM herbs, and bioactive compounds derived from TCM in exerting anti-OP effects through neuropeptide modulation. These studies highlight the multi-targeted and multi-mechanistic pharmacological actions of TCM in treating OP. By integrating these findings, we aim to enhance the understanding of neuropeptides’ roles in bone metabolism and to explore the development of neuropeptide-targeted TCM therapies for OP management. This comprehensive perspective highlights the potential of neuropeptides as therapeutic targets, paving the way for innovative approaches to treating OP.

## 1 Introduction

Osteoporosis (OP) is a systemic bone metabolic disorder characterized by reduced bone strength and an increased risk of fractures, primarily resulting from decreased bone mass and the deterioration of bone microstructure. Globally, OP is estimated to affect approximately 18.3% of the population, with a notably higher prevalence among women at 23.1% compared to at 11.7% among men. In the aging demographic, this figure soars to 35.3% (Salari et al., 2021), representing a significant burden on healthcare systems, economies, and societies at large. The root cause of OP lies in the imbalance between bone formation and resorption, where insufficient bone formation fails to counteract excessive bone resorption (Li et al., 2021a). Bone formation is commonly associated with bone mesenchymal stem cells and osteoblasts, while osteoclasts are responsible for bone resorption. Consequently, the dynamic interplay between these cell types determines both the quality and quantity of bone. Studies have highlighted that signaling pathways, methylation modifications, and non-coding RNAs mediate these processes, along with the presence of endogenous active substances in the microenvironment.

Neuropeptides, the largest and most diverse class of signaling molecules in the brain, play multifaceted roles beyond neurotransmission. They can function as neurotransmitters, modulate ongoing neurotransmission by other transmitters, act as autocrine or paracrine regulators within localized cellular environments, and serve as hormones over the long term (Wilkinson and Brown, 2015). Neuropeptides exhibit high activity and a broad spectrum of effects, involving in the modulation of social valence, sleep, appetite, anxiety, stress response, pain perception (Smith et al., 2020; Wang et al., 2018). Research has indicated that neuropeptides regulate bone turnover and endogenous levels in the skeleton, influencing the occurrence and progression of OP (Chen et al., 2023; Wang et al., 2021). Specifically, Neuropeptide Y (NPY), Vasoactive Intestinal Peptide (VIP), Calcitonin Gene-Related Peptide (CGRP), and Substance P (SP), along with their respective receptors, have been identified as being expressed in both brain and bone tissue, as key contributors to bone growth and development (Liu et al., 2018a). Their absence can lead to bone metabolism imbalance and bone mass loss. These findings highlight the significant regulatory role of neuropeptides in bone metabolism.

In Traditional Chinese Medicine (TCM), the primary pathogenesis of OP is attributed to kidney deficiency, blood stasis, and spleen deficiency. Treatment focuses on nourishing the kidney and spleen, promoting blood circulation, and resolving blood stasis (Cao et al., 2024). TCM is widely used in the management of OP, recognized for its safety and efficacy. Clinical trials have shown that TCM can improve bone mineral density (BMD), alleviate pain, and cause minimal side effects in OP patients (Jia et al., 2022; Li et al., 2022a). For example, a randomized controlled trial involving 200 patients demonstrated that Zuogui and Yougui pills significantly improved lumbar spine and femoral BMD, reduced pain, and enhanced quality of life (Li et al., 2018). Similarly, our previous study revealed that Yigu capsules increased lumbar and hip BMD, relieved ostealgia, and extended motion time without causing new fractures or adverse reactions (Zhang et al., 2004). Unlike conventional treatments, TCM takes a holistic approach, addressing systemic imbalances and targeting multiple pathways. This approach facilitates personalized treatments with fewer adverse effects, aiming to optimize bone metabolism and overall health (Cao et al., 2024). Recent research highlights TCM’s multifaceted mechanisms in preserving and enhancing bone health. Specifically, TCM stimulates osteoblast activity (essential for bone formation) and inhibits osteoclast function (reducing bone resorption), maintaining the balance between bone formation and resorption. These effects are partly mediated by neuropeptides, suggesting that TCM may act as a neuropeptide modulator (Cao et al., 2024; Lei et al., 2021; Li et al., 2023; Peng et al., 2022).

To date, research publications on neuropeptides have exceeded 2,600, with sources from the Chinese National Knowledge Infrastructure (http://www.cnki.net/), the National Science and Technology Library (http://www.nstl.gov.cn/), and approximately 1,253 from the PubMed (www.pubmed.gov) database. This review aims to enhance the understanding of the role of neuropeptides in bone metabolism and explore potential anti-OP strategies using TCM based on neuropeptide targets. More than 70 references were consulted from various databases, spanning the period from their inception to October 2024. These findings highlight the increasing number of therapeutic approaches.

## 2 The role of neuropeptides in bone metabolism

**FIGURE 1:**
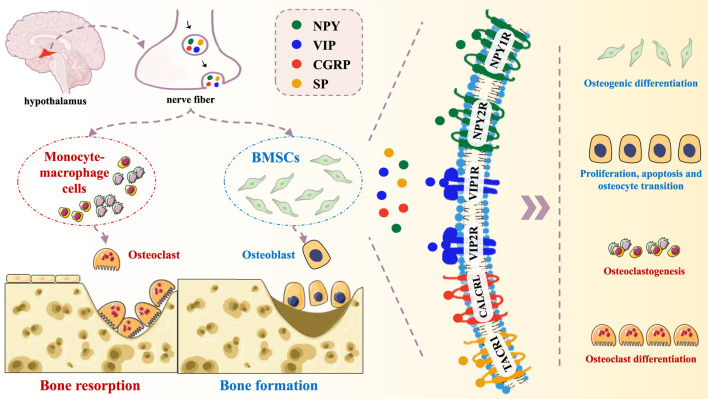
Schematic representation of neuropeptide mechanisms in bone metabolism.

Until now, synapses have not been identified within bone; however, various neuropeptides have been found in bone tissue, including NPY, VIP, SP and CGRP. Neuropeptides commonly released into the extracellular space through non-synaptic vesicular fusion within axon varicosities. After their release into the extracellular fluid, these signaling molecules are transported to receptors on the targeted bone tissues via energy gradients, subsequently stimulating the activities of related cells (Figure 1; Table 1).

**TABLE 1:** Role of Neuropeptides in bone metabolism.

Name	Model	Effect	References
NPY	*NPY* knockout mice	Trabecular bone and bone volume were increased in NPY knockout mice	Baldock et al. (2009)
	*NPY* hypothalamus specific overexpression mice	Bone mass was reduced in cases of NPY overexpression in the hypothalamus	Baldock et al. (2009)
	*NPY* knockout mice	Global deletion of NPY resulted in a reduced femoral cortical cross-sectional area and decreased bone strength in this model	Wee et al. (2019)
	Mouse bone marrow cells	NPY inhibits isoprenaline-induced osteoclastogenesis by suppressing agonist-driven increases in cAMP and RANKL production in mouse bone marrow cells	Amano et al. (2007)
NPY1R	Conditional knockout *NPY1R* in osteoblasts	Conditional knockout of NPY1R in osteoblasts increases bone mineral deposition and bone formation rates	Baldock et al. (2007), Lee et al. (2011)
	*NPY1R* knockout mice	The knockout of NPY1R was found to enhance osteoblast activity, increase bone turnover, and elevate bone mass	Baldock et al. (2009), Liu et al. (2016a)
	Bone mesenchymal stem cells	Overexpressed NPY1R inhibits the osteogenic differentiation of BMSCs via the cAMP/PKA/CREB signaling pathway	Xie et al. (2020a)
	Bone mesenchymal stem cells	NPY1R antagonist promotes osteogenic differentiation of BMSCs and bone repair	Liu et al. (2016a)
	MC3T3-E1 cell line	*NPY1R* antagonist BIBP3226 promotes extracellular signal-regulated kinases phosphorylation and osteoblast differentiation	Yu et al. (2016)
	Rats undergoing fracture healing stage	The expression of NPY1R was very low at the early healing stage, but it increased at the middle stage and then decreased at the late stage	Zou et al. (2020)
	*NPY1R* knockout mice	Knockout of *NPY1R* lead to a greater bone strength	Sousa et al. (2020)
NPY2R	Ovariectomized (OVX) mice	NPY2R antagonist promotes bone mineral density, increases bone volume within trabecular regions and greater trabecular number, enhances mineralization, and reduces bone loss	Seldeen et al. (2018)
	*NPY2R*-deficient mice	*NPY2R*-deficient mice’s trabecular bone volume, number, and thickness increased, stimulating bone mineralization and formation	Baldock et al. (2002)
	Conditional knockdown peripheral *NPY2R* mice	It protects against diet-induced obesity, reduces weight gain, and improves glucose tolerance, without any adverse effect on lean mass or bone	Shi et al. (2011)
	*NPY2R*-deficient mice	Delete *NPY2R* resulting in a doubling of trabecular bone volume	Lundberg et al. (2007)
VIP	Postmenopausal osteoporosis patients	VIP was decreased in the serum, and the content of VIP in serum was positively associated with BMD at the femoral neck, lumbar spine 1–4, and total hip	Wang et al. (2019)
	De-sympathetic rats	Sympathetic VIP secretion could lead to an increase in the number of osteoclasts and the levels of cortical bone resorption on the surface of the mandible in rats	Hill et al. (1991)
	Osteosarcoma cell line ROS 17/2.8	VIP represses osteoclast differentiation without affecting the number of osteoclast precursor cells	Qu et al. (2021)
	Osteoclasts	VIP promotes the secretion of bone protective proteins from bone marrow stromal cells or osteoblasts, inhibits the expression of RANKL and nuclear factor kappa B receptor activator (RANK), and thereby indirectly inhibits the differentiation of osteoclasts and bone resorption	Mukohyama et al. (2000)
	Osteoblasts	VIP accelerates the formation of mineralized nodules of osteoblasts and promotes osteogenic differentiation	Lundberg et al. (1999)
	Bone mesenchymal stem cells	VIP activates the Wnt/β-catenin signaling pathway to enhance BMSCs and stimulate the expression of vascular endothelial growth factor to promote angiogenesis	Shi et al. (2020)
	Osteoblasts	VIP released from skeletal nerve endings could induce the proliferation and activity of osteoblasts via enhancing junctional intercellular communication between cells, and further influence bone formation	Ma et al. (2013)
VIP1R VIP2R	Periosteum-derived osteoblastic cells and Osteosarcoma-derived cells	Osteoblasts derived from periosteum and osteosarcoma expressed VIP1R but not VIP2R	Togari et al. (1997)
	Osteoblasts	The expression of VIP1R increased during the late stage of osteogenic differentiation, suggesting a close correlation between VIP1R and bone formation	Lundberg et al. (2001)
	Osteoblasts	Activation of VIP2R increases the RANKL/OPG ratio and the expression of IL-6 by activating signaling pathways, such as cAMP-ERK, p44/p42 MAPK, and cAMP/PKA/CREB.	Natsume et al. (2010), Persson and Lerner (2011), Persson et al. (2005)
CGRP	OVX rats	CGRP level is upregulated in the spinal cord while downregulated in serum and femoral tissue	Zhang et al. (2021)
	αCGRP-deficient mice	αCGRP-deficient resulted in a significant reduction of osteoblast number, incomplete healing of the callus, and high rates of nonunion in this model	Appelt et al. (2020)
	BMSCs isolated from female osteoporotic rats	CGRP reverses the decrease in proliferation and differentiation capabilities of BMSCs from osteoporotic rats, promoting their proliferation and migration through the Wnt/β-catenin signaling pathway, inducing osteogenic differentiation and mineralization	Liang et al. (2015)
	OVX rats	CGRP stimulates the osteoblasts to produce insulin-like growth factor 1 (IGF-1) while inhibiting the generation of tumor necrosis factor-α (TNF-α) in this model	Valentijn et al. (1997)
	Osteoblasts	CGRP increases both the expression and the levels of cytoplasmic β-catenin by binding to its receptor, and inhibit human osteoblasts apoptosis stimulated by dexamethasone or by serum deprivation in this model	Mrak et al. (2010)
CALCRL	OVX rats	The expression of CALCRL is decreased in the brain, while increased in the femora of OVX rats	Liu et al. (2018a)
	Rats undergoing fracture healing	Overexpression of the *CALCRL* gene would increase bone mass in the femora of rats while silencing it would exhibit the opposite trend in this model	Zhang et al. (2016)
	Mouse induced pluripotent stem cells	CALCRL is expressed at all stages of cell differentiation, including the pluripotent stem cell stage, with peak expression occurring at the early osteoblastic differentiation stage in this model	Nagao et al. (2014)
	OHS-4 osteoblastic cells	CGRP increases intracellular free Ca^2+^ concentration but is not coupled to adenylate cyclase in CALCRL-positive OHS-4 osteosarcoma cells	Drissi et al. (1999)
	*CALCRL*-deficient monocyte/macrophage cells	*CALCRL*-deficient monocyte-macrophage cells could not develop into mature osteoclasts after RANKL stimulation	Cho et al. (2022)
SP	Femoral shaft fracture in OVX mice	At the fracture site, SP decreased significantly at all time points, and its level at an early stage was higher than later stage in this model	Ding et al. (2010)
	OVX rats	Pretreatment with SP could block type H vessel loss, accompanied by the enrichment of nitric oxide and sustained angiogenic factors	Kim et al. (2023)
	OVX rats	SP ameliorates chronic inflammation by promoting Treg cell polarization and inhibiting the development of osteoclastogenic Th17 cells, rejuvenates stem cells, and enables stem cells to repopulate and differentiate into osteoblasts in this model	Piao et al. (2020)
	OVX mice	Application of L-703606 inhibits recruitment of BMSCs to bone remodeling sites, which was evidenced by the increased number of osteoclasts, decreased number of osteoblasts, and increased osteoid volume in the secondary spongiossa in this model	Zheng et al. (2016)
	BMSCs	SP stimulates cell proliferation, inhibits osteogenic differentiation and these effect would be inhibited by adding an SP antagonist in this model	Liu et al. (2016b)
	Osteoblasts precursors and osteoclasts precursors	SP stimulates the proliferation of BMSCs in a concentration-dependent manner. It behaved as stimulated alkaline phosphatase and osteocalcin expression, increased alkaline phosphatase activity, and upregulated Runx2 protein level	Wang et al. (2009)
	monocyte/macrophage cell line	SP induces the osteoclastic differentiation of monocyte/macrophage cell precursor cells by up-regulating the expression of NF-κB and TNF-α, thereby promoting bone resorption	Lam et al. (2000)
	Tachykinin (Tac) 1-deficient mice	The absence of SP results in a slight reduction of bone resorption rate but concomitantly in a critical reduction of bone formation and mineralization rate in this model	Niedermair et al. (2018)
TACR1	OVX rats	In the OVX rat, TACR1 decreased in the brain tissue but increased TACR1 in the bone tissue	Liu et al. (2018b)
	Osteoblasts	TACR1 promotes the osteogenic differentiation of late-stage osteoblasts, while blocking TACR1 could achieve the opposite effect	Goto et al. (2007)

### 2.1 NPY and its receptors regulate bone metabolism in both animal and cellular models

NPY, a 36-amino acid polypeptide, belongs to the pancreatic polypeptide family and is widely distributed throughout both the central and peripheral nervous systems. In the central nervous system, NPY is particularly abundant in several key regions, including the hypothalamus, cerebral cortex, brainstem, striatum, and limbic system, with especially high concentrations found in the arcuate nucleus of the hypothalamus. In the peripheral nervous system, NPY plays a crucial role in the sympathetic nervous system, where it is co-stored with norepinephrine in sympathetic neurons and released alongside norepinephrine upon neural stimulation (Baldock et al., 2009; Shende and Desai, 2020). The role of NPY and its receptors, specifically the Neuropeptide Y1 receptor (NPY1R) and the Neuropeptide Y2 receptor (NPY2R), is closely linked to the pathogenesis of OP, suggesting that NPY signaling may significantly influence bone metabolism (Khor and Baldock, 2012).

#### 2.1.1 NPY and bone metabolism

NPY functions as a regulator of bone homeostasis, with its effects on bone mass closely associated with fluctuations in hypothalamic NPY levels and energy intake. Research has demonstrated that in ovariectomized (OVX) rats, a decrease in bone density coincides with a significant increase in NPY expression in both the hypothalamus and femur (Li et al., 2022b). In NPY knockout (KO) mouse, both trabecular and cortical bone volumes are elevated. Furthermore, reducing NPY expression in the hypothalamus has been shown to enhance bone mass in rats with adequate energy intake. Conversely, NPY expression in the hypothalamus decreases during fasting, which is accompanied by a reduction in bone mass, likely linked to decreased energy consumption (Baldock et al., 2009).

Additionally, NPY is expressed in osteoblasts, bone marrow mesenchymal stem cells (BMSCs), and osteoclasts, and it is directly involved in the differentiation and proliferation of these cells. Research has shown that NPY can inhibit the expression of markers associated with osteoblast differentiation and suppress the differentiation of osteoblasts (Baldock et al., 2009). The specific knockout of NPY derived from BMSCs has been found to enhance cortical bone in KO rats, promoting the expression of bone sialoprotein and osteocalcin in BMSCs, thereby facilitating the osteogenic differentiation of these cells (Wee et al., 2019; Wee et al., 2020). Other studies suggest that NPY may also be involved in regulating bone resorption. NPY can inhibit the cAMP/PKA pathway activated by isoproterenol, which promotes the secretion of receptor activator of nuclear factor-κB ligand (RANKL) by osteoblasts, consequently increasing the bone resorption activity of osteoclasts (Amano et al., 2007). In summary, with adequate energy intake, the inhibition of NPY expression of NPY in bone tissue leads to an increase in bone mass and enhances the processes of bone healing. In conclusion, the mechanisms by which NPY influences OP may involve promoting osteogenic differentiation in BMSCs and inhibiting bone resorption in osteoclasts.

#### 2.1.2 NPY receptors and their effects on bone metabolism

NPY1R is expressed in both central and peripheral tissues, demonstrating an inverse correlation in its expression levels between the central nervous system and bone tissues in OP model animals. This makes NPY1R an important target for regulating bone metabolism. Studies indicate that NPY1R expression is elevated in the bone tissue of OVX rats, while its expression decreases in the central nervous system. The administration of NPY1R could mitigate bone tissue damage caused by the OVX operation (Xie et al., 2020a). Baldock et al. found that NPY1R KO rats exhibit increased bone mass. Further investigations revealed that the specific knockout of NPY1R in the hypothalamus does not affect bone mass, whereas the specific knockout of NPY1R in osteoblasts enhances the rates of bone mineral deposition and bone formation, thereby increasing bone mass in mouse (Baldock et al., 2007; Lee et al., 2011). NPY1R also regulates the healing process of fractures. Zou et al. found that during the initial stage of fracture healing, the expression level of NPY1R is relatively low. However, it increases significantly during the intermediate stage and then declines back to a lower level in the later stage. This pattern indicates that NPY1R is involved in the mid-to-late stages of fracture healing and aids in the formation and remodeling of callus tissue (Zou et al., 2020). Recent studies have shown that NPY1R deficiency can enhance bone strength and reduce fracture risk by improving the ultrastructure of the extracellular matrix and increasing matrix maturity (Sousa et al., 2020).

Research indicates that the knockout of *NPY1R* gene not only enhances the activity of osteoblasts in mouse, increasing bone turnover and bone mass, but also promotes the proliferation and osteogenic differentiation of BMSCs (Baldock et al., 2009; Liu et al., 2016b). Further exploration has revealed that activating NPY1R expression can inhibit the osteogenic differentiation of BMSCs via the cAMP/PKA/CREB signaling pathway (Xie et al., 2020b), whereas inhibiting NPY1R expression enhances the osteogenic differentiation capabilities of BMSCs (Liu et al., 2018a). Similarly, inhibiting the expression of NPY1R on pre-osteoblastic MC3T3-E1 can also enhance osteogenic differentiation, with the underlying mechanism linked to the regulation of the extracellular signal-regulated kinase signaling pathway (Yu et al., 2016). Thus, NPY1R is expressed in osteoblasts on both cortical and trabecular bone surfaces, and its inhibition of osteoblast and BMSC may be related to the regulation of NPY. Additionally, Dong et al. suggested that regulating the NPY/NPY1R signaling pathway promotes osteogenic differentiation of BMSCs and fracture healing (Dong et al., 2018).

NPY2R expression increases in the tibia and dorsal root ganglia of OVX rats. The administration of NPY2R antagonists in OVX rats can enhance bone density and mitigate bone loss (Seldeen et al., 2018). In contrast to NPY1R, both global and conditional knockout of NPY2R in the hypothalamus result in an increased number of trabecular bones in the femurs of rats (Baldock et al., 2002). However, the conditional knockout of peripheral NPY2R has no effect on the skeletal system (Shi et al., 2011). Therefore, hypothalamic NPY2R might play a more critical role in regulating bone metabolism compared to peripheral NPY2R. Furthermore, unlike other receptors, NPY2R functions as an auto-receptor, providing negative feedback regulation of NPY expression. The conditional knockout of NPY2R on NPY neurons in the hypothalamus of rats leads to an increase in NPY levels within the hypothalamus, which diminishes the stimulatory effect on bone formation and may even inhibit it (Hohmann et al., 1986). Thus, apart from NPY2R on NPY neurons, NPY2R on other neurons in the hypothalamus may play a significant role in regulating bone metabolism.

Moreover, promoting bone formation following NPY2R knockout may be associated with an increased osteogenic differentiation of osteoblasts and the proliferation of BMSCs (Baldock et al., 2002). Lundberg et al. found that the number of BMSCs in rats with systemic NPY2R deficiency increased nearly doubled in global NPY2R knockout mice, and NPY2R knockout led to a decrease in NPY1R expression on osteoblasts (Lundberg et al., 2007). Therefore, in addition to the role of NPY2R expressed on NPY neurons, NPY2R on other hypothalamic neurons may also significantly contribute to the regulation of bone metabolism.

### 2.2 VIP and its receptors regulate the process of bone metabolism in both animal and cellular models

VIP, composed of 28 amino acids, is a small neuropeptide that belongs to the glucagon-like polypeptide family. It is primarily released by intestinal neurons, as well as by endocrine and immune cells. The main receptors for VIP are vasoactive intestinal peptide receptor 1 (VIPR1) and vasoactive intestinal peptide receptor 2 (VIPR2). In bone tissue, VIP is predominantly found in the parasympathetic neurons of the skeleton, with smaller amounts present in postganglionic sympathetic neurons and primary sensory neurons. VIP plays a crucial role in regulating bone metabolic balance (Hohmann et al., 1986).

#### 2.2.1 VIP and bone metabolism

VIP plays a dual role in inhibiting bone resorption and promoting bone formation. Clinical studies have demonstrated that serum levels of VIP are inversely correlated with the severity of postmenopausal OP (Wang et al., 2019), indicating a significant relationship between VIP and bone resorption. Research suggests that inhibiting sympathetic VIP secretion can lead to an increase in the number of osteoclasts and elevated levels of cortical bone resorption on the surface of the mandible in rats (Hill et al., 1991; Joo et al., 2004). Furthermore, VIP can promote osteoclast activity without affecting the number of osteoclast precursors (Qu et al., 2021). VIP not only acts directly on osteoclasts but also indirectly regulates bone resorption by influencing osteoblasts. *In vitro* studies by Mukohyama et al. have shown that VIP can enhance the secretion of bone protective proteins from BMSCs or osteoblasts, inhibit the expression of nuclear factor kappa B receptor activator (RANK) and RANKL (receptor activator of RANK), and thereby indirectly inhibit osteoclasts differentiation and bone resorption (Mukohyama et al., 2000). Additionally, VIP accelerates the formation of mineralized nodules in osteoblasts, promoting osteogenic differentiation (Lundberg et al., 1999; Ma et al., 2013). Shi et al. has found that VIP can activate the Wnt/β-catenin signaling pathway to enhance BMSCs and stimulate the expression of vascular endothelial growth factor to promote angiogenesis (Shi et al., 2020).

#### 2.2.2 VIP receptors and their effects on bone metabolism

VIP1R and VIP2R are the primary receptors for VIP. VIP1R is closely associated with the osteogenic differentiation of osteoblasts, whereas VIP2R is involved in both bone resorption and formation. Studies have demonstrated that osteoblasts derived from the periosteum and osteosarcoma express VIP1R but not VIP2R (Togari et al., 1997). The expression of VIP1R increases during the later stages of osteoblast differentiation, indicating a strong correlation between VIP1R and bone formation (Lundberg et al., 2001). In contrast, its activation can elevate the RANKL/OPG ratio and increase the expression of interleukin-6 by activating various signaling pathways, including cAMP-ERK, p44/p42 MAPK, and cAMP/PKA/CREB (Natsume et al., 2010; Persson and Lerner, 2011; Persson et al., 2005), thereby promoting osteoclast-mediated bone resorption. Furthermore, the activation of VIP2R can enhance the levels of cyclic adenosine monophosphate (cAMP) in osteoblasts, stimulating the expression of alkaline phosphatase and osteocalcin, which in turn promotes osteogenic differentiation (Lundberg et al., 1999; Ma et al., 2013).

### 2.3 CGRP and its receptors regulate the process of bone metabolism in both animal and cellular models

CGRP, a neuropeptide consisting of 37 amino acids, is released by sensory nerve terminals and is widely distributed throughout both the central and peripheral nervous systems. Research has shown that CGRP is primarily localized in the periosteum and bone marrow. It can bind to the calcitonin receptor-like protein (CALCRL) on bone-related cells, thereby influencing their activity and playing a crucial regulatory role in bone growth and repair (Irie et al., 2002).

#### 2.3.1 CGRP and bone metabolism

CGRP has significant benefits for bone growth and repair. In OVX rats, CGRP levels are upregulated in the spinal cord but downregulated in the serum and femoral tissue (Zhang et al., 2021). In a rat model of femoral fracture, α-CGRP deficiency leads to severe impairments in bone regeneration, characterized by a significant reduction in osteoblast numbers, incomplete healing of the callus, and high rates of nonunion. These impairments are closely associated with the differential expression of specific genes related to ossification, bone remodeling, and adipogenesis. Among these, CGRP-induced peroxisome proliferator-activated receptor gamma (PPAR-γ) signaling plays a pivotal role in fracture healing (Appelt et al., 2020). CGRP may enhance the proliferation and migration of BMSCs extracted from OP rats, via the Wnt/β-catenin signaling pathway (Liang et al., 2015). CGRP stimulates the homing and differentiating into osteoblasts. Subsequently, these osteoblasts produce increased levels of insulin-like growth factor 1 (IGF-1) while inhibiting the generation of tumor necrosis factor-α (TNF-α), thereby further regulating osteoblast function (Valentijn et al., 1997). Additional studies have demonstrated that CGRP enhances bone formation mediated by osteoblasts by stimulating Wnt signaling, promoting the expression of bone morphogenetic protein 2 (BMP-2) and Runt-related transcription factor 2 (Runx2), inhibiting the expression of inflammatory factors, and preventing apoptosis (Cai et al., 2016; Mrak et al., 2010; Tuzmen and Campbell, 2018). Consequently, the effects of CGRP on bone metabolism are primarily related to the regulation of BMSCs and osteoblasts.

#### 2.3.2 CGRP receptors and their effects on bone metabolism

CALCRL is a known receptor for CGRP. Its expression is decreased in brains of OVX rats, demonstrating a negative correlation with bone formation (Liu et al., 2018b). Overexpression of the *CALCRL* gene enhances bone mass in the femora of rats, whereas silencing the *CALCRL* gene exhibits the opposite effect (Zhang et al., 2016). Research has also indicated that CALCRL is expressed in bone-related cells, including induced pluripotent stem cells, hematopoietic precursor cells, and BMSCs, which are closely linked to their differentiation processes. CALCRL and receptor activity-modifying protein one form a functional heterodimer complex that can be activated by CGRP, leading to the expression of the *Creb1* gene and the osteoblast-specific gene Sp7, thereby facilitating the osteogenic differentiation of periosteal-derived stem cells (Zhang et al., 2016). CALCRL expression increases during the osteogenic differentiation of induced pluripotent stem cells and periosteal-derived osteoblasts, participating in the local regulation of human bone metabolism alongside norepinephrine (Nagao et al., 2014; Wang et al., 2011; Zhang B. et al., 2022). Furthermore, CGRP can promote the osteogenic differentiation of osteosarcoma cells, which is associated with an increase in intracellular free calcium ion concentration (Drissi et al., 1999). Additionally, the *CALCRL* gene has an inhibitory effect on bone resorption. Studies have shown that monocyte-macrophage cells with *CALCRL* gene defects cannot mature into mature osteoclasts following after RANKL stimulation (Cho et al., 2022).

### 2.4 SP and its receptors regulate the process of bone metabolism in both animal and cellular models

SP is a neuropeptide located in primary sensory neurons and is released from the axons of these neurons following neural stimulation. It belongs to the tachykinin family and is primarily synthesized by neurons in the dorsal root ganglion. SP binds to the neurokinin-1 receptor to exert various physiological effects. Research has demonstrated that SP is present in bones, bone marrow, epiphyseal plates, ligaments, and muscles, where it is distributed in active sites of bone formation during bone metabolism. It serves as a crucial local regulator of bone-related cellular functions (Liu et al., 2007).

#### 2.4.1 SP and bone metabolism

SP can delay the onset and progression of OP while promoting bone repair. In OVX rat models, the expression of SP in bone tissue is significantly reduced (Liu X. et al., 2018). Similarly, in the OVX fracture rat model, the fracture healing rate in OVX rats is notably decreased, which is accompanied by a reduction in SP content at the fracture site (Ding et al., 2010). Conversely, SP intervention can protect against the loss of H-type blood vessels in early estrogen-deficient bone tissue by regulating oxidative stress, thereby preventing the development of OP (Kim et al., 2023). Furthermore, research indicates that SP can mitigate chronic inflammation by promoting the polarization of regulatory T cell and inhibiting the development of T helper 17 cells, which are associated with osteoclast generation and activation. This process enhances the proliferation and osteoclastogenesis of BMSCs from OVX rats, thereby accelerating bone formation (Piao et al., 2020). Inhibition of SP signaling not only reduces bone mass in normal rats but also accelerates bone loss in OVX rats (Zheng et al., 2016). Furthermore, SP promotes the proliferation and osteogenic differentiation of BMSCs or osteoblasts, with its effects dependent on the concentration of SP. *In vitro* cultures of BMSCs, both the number and size of bone marrow cell colonies are positively correlated with SP concentration, indicating that it stimulates osteogenic differentiation (Shih and Bernard, 1997). Additionally, SP enhances the proliferation of osteoblasts in rat models of spinal cord injury through the RANKL/OPG signaling axis; however, it inhibits their osteogenic differentiation and mineralization (Liu et al., 2016a).

Other studies suggest that low concentrations of SP can enhance the proliferation of BMSCs, while high concentrations of SP have the opposite effect (Wang et al., 2009). Furthermore, SP induction can accelerate bone resorption by osteoclasts, with the underlying mechanisms linked to inflammatory responses. SP can induce the osteoclastic differentiation of RAW 264.7 cells through the upregulation of NF-κB and TNF-α, thereby promoting bone resorption (Lam et al., 2000; Wang et al., 2009). A deficiency of SP may reduce the number of osteoclasts and subsequently diminish the capacity for bone resorption (Niedermair et al., 2018). In summary, SP promotes the bone resorption activity of osteoclasts and exhibits beneficial effects on bone repair in OP animal models. The underlying mechanism may involve an acceleration of the bone remodeling rate by SP, with its effects on bone formation outweighing its effects on bone resorption.

#### 2.4.2 SP receptors and their effects on bone metabolism

TACR1 is a well-known receptor for SP, and it is involved in the mediation of phosphatidylinositol metabolism of SP. In OVX rats, the expression of TACR1 was also significantly reduced in the brain, but increased in the bone (Liu et al., 2018a). Further studies have demonstrated that activating TACR1 can promote the osteogenic differentiation of late-stage osteoblasts, while blocking TACR1 produces the opposite effect (Goto et al., 2007). Therefore, TACR1 expressed on osteoblasts presents a promising therapeutic target for the treatment of OP.

## 3 The role of traditional Chinese medicine in modulating bone metabolism through neuropeptide regulation

**FIGURE 2:**
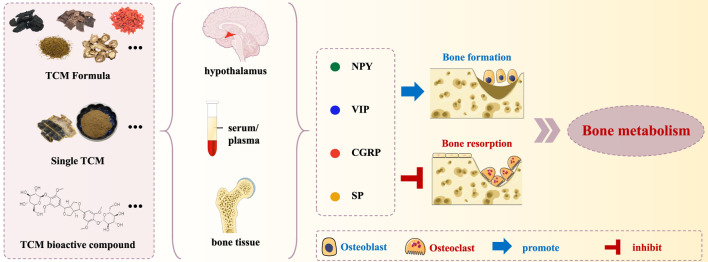
Visual summary of TCM’s regulatory effects on neuropeptides.

Neuropeptides are highly susceptible to degradation, presenting significant challenges for their application and stability in both *in vivo* and *in vitro* studies. This instability often limits their therapeutic potential and necessitates alternative strategies to harness their benefits. TCM has demonstrated a significant role in combating OP by promoting the secretion of neuropeptides in both bone and brain tissues. These neuropeptides, in turn, modulate the activity of bone-related cells, such as osteoblasts and osteoclasts, which are critical for maintaining bone homeostasis. By enhancing neuropeptide production, TCM indirectly supports bone formation while inhibiting bone resorption, contributing to the preservation of bone density and structural integrity (Figure 2; Table 2). This unique mechanism suggests that TCM may serve as a valuable adjunct in OP treatment, addressing challenges associated with neuropeptide degradation while offering a holistic and multifaceted approach to bone health.

**TABLE 2:** The role of traditional Chinese medicine in modulating bone metabolism through neuropeptide regulation.

Traditional Chinese medicine		Models	Neuropeptides	Effects	References
TCM Formula	Yigu Capsule	Ovariectomized SD rats	NPY	Yigu Capsule decreases the protein and mRNA expression of NPY in plasma and bone tissue while increasing bone mineral density	Zhu et al. (2015)
		Ovariectomized SD rats	CGRP SP	Yigu Capsule increases the levels of CGRP and SP in plasma and bone tissue, as well as enhances bone mineral density	Zhu et al. (2012)
	Zuogui Pill	Ovariectomized SD rats	NPY VIP CGRP SP	Zuogui Pill elevates the expression of NPY, VIP, CGRP, and SP in BMSCs and bone tissue, concurrently increasing bone mineral density	Yang et al. (2016)
	Yougui Pill	Ovariectomized SD rats	NPY VIP CGRP SP	Yougui Pill enhances the expression of NPY, VIP, CGRP, and SP in BMSCs and bone tissue, simultaneously improving bone density	Yang et al. (2016)
		Ovariectomized SD rats	NPY VIP	Yougui Pill raises the expression levels of NPY and VIP in bone tissue and enhances bone mineral density	Chen et al. (2017)
	Zhuangjing Xugu Decoction	Fracture model in male Wistar rats	NPY	Zhuangjing Xugu Decoction promotes the expression of bone morphogenetic protein-7 (BMP-7) and NPY in fibroblasts and osteoblasts within the fracture callus	Wang et al. (2013)
	Bushen Huatan Decoction	Ovariectomized SD rats	NPY	Bushen Huatan Decoction reduces NPY protein expression in the hypothalamus, regulates abnormal lipid metabolism through the leptin-hypothalamic NPY axis, and mitigates osteoporosis	Zhang et al. (2022b)
	Zhuanggu Zhitong Decoction	Ovariectomized SD rats	NPY	Zhuanggu Zhitong Decoction decreases NPY content in the hypothalamus, inhibits NPY secretion, and promotes bone formation	Yi et al. (2016)
	Bushen Jianpi Decoction	Ovariectomized SD rats	VIP	Bushen Jianpi Decoction reduces VIP levels in peripheral blood serum, increases osteoprotegerin content, and decreases the level of osteoclast development-activating factors such as receptor activator of nuclear factor κB ligand	Li et al. (2014)
	Erxian Decoction	Spinal cord injury male SD rats	SP	Erxian Decoction reduces the positive expression of SP in bone cells, osteoblasts, osteoclasts, and bone marrow stromal cells within trabecular and cortical bone, alleviating osteoporosis following spinal cord injury	Wu et al. (2015)
Single TCM	*Plastrum testudinis*	Ovariectomized SD rats	NPY NPY1R NPY2R	*Plastrum testudinis* downregulates the mRNA expression of NPY, NPY1R, and NPY2R in the whole brain and hypothalamus, promotes bone formation, and reduces the mRNA expression of NPY in the femur. It also downregulates the protein expression of NPY and NPY1R in the whole brain, decreases NPY1R protein expression in the hypothalamus, and lowers NPY protein levels in the femur. Additionally, it increases the protein expression of NPY1R and NPY2R in the femur and promotes the mRNA and protein expression of bone formation-related markers such as ALP, BMP-2, COL1, and Runx2	Li et al. (2022a)
	*Epimedium brevicornu Maxim*	Ovariectomized SD rats	NPY NPY1R NPY2R VIP VIP1R VIP2R CGRP CALCRL SP TACR1	*Epimedium brevicornu Maxim.* increases the expression of NPY, CGRP, VIP, and VIP2R in bone tissue, while reducing the mRNA expression of NPY1R, NPY2R, and TACR1. It also elevates protein levels of CRLR, VIP, VIP2R, and SP in bone tissue while decreasing NPY1R and TACR1 protein levels	Liu et al. (2018a)
TCM bioactive compound	Eleutheroside E	Ovariectomized C57BL6/J mice	NPY VIP CGRP SP	Eleutheroside E increases the levels of NPY, VIP, CGRP, and SP in serum, decreases tartrate-resistant acid phosphatase levels, enhances the content of bone formation marker I procollagen amino-terminal peptide, and reduces TNF-α, IL-6, and IL-10 levels	Zhou et al. (2023)
	Loganin	Ovariectomized C57BL6/J mice	NPY VIP CGRP SP	Loganin promotes the expression of NPY, VIP, CGRP, SP, and P1NP in serum, decreases levels of TRAP, TNF-α, and IL-6, moderates intestinal flora imbalances, and subsequently promotes bone formation while inhibiting bone resorption	Xie et al. (2024)

### 3.1 Traditional Chinese medicine formula

The role of TCM in enhancing bone health and treating OP has been extensively studied. Among these, the Yigu Capsule has demonstrated the ability to increase bone density in OVX rats, accompanied by elevated NPY levels in both plasma and bone tissue (Zhu et al., 2015). Similarly, the Yougui Pill has been found to significantly enhance NPY levels in the bone tissue, contributing to improvements in bone mineral density in OVX rats (Chen et al., 2017). The Zhuangjing Xugu Decoction, on the other hand, facilitates fracture healing by upregulating NPY expression in callus tissue fibroblasts (Wang et al., 2013). Furthermore, the Bushen Huatan Decoction has been reported to modulate the dysregulated adipose-bone metabolism in OVX rats via the hypothalamic leptin-NPY axis (Zhang Y. et al., 2022). Meanwhile, the Zhuanggu Zhitong Decoction has demonstrated the ability to reduce hypothalamic NPY levels while concurrently increasing bone density in OP rats (Yi et al., 2016).

In addition to NPY, other neuropeptides have been implicated in bone health. The Zuogui Pill and Yougui Pill enhance bone repair by increasing the levels of VIP in BMSCs and bone tissue of OVX rats (Yang et al., 2016). Similarly, the Bushen Jianpi Decoction elevates bone mineral density and VIP levels in the peripheral blood serum of OP rats (Li et al., 2014).

The CGRP is another key factor influenced by traditional medicines. The Yigu Capsule improves bone mineral density in OVX rats by elevating CGRP levels in bone tissue and plasma, thus offering a potent therapeutic approach for OP (Zhu et al., 2012). In a similar vein, the Zuogui Pill and Yougui Pill foster bone repair by increasing CGRP levels in BMSCs and the bone tissue of OVX rats (Yang et al., 2016).

Moreover, SP has been identified as a critical mediator of bone repair. Both the Zuogui Pill and Yougui Pill promote bone repair and stimulate BMSC proliferation in OVX rats by increasing SP content in bone tissue (Yang et al., 2016). The Yigu Capsule also raises SP levels in bone tissue and plasma, leading to increased bone density (Zhu et al., 2012). Finally, the Erxian Decoction enhances bone density through the upregulation of SP expression in the distal femur of OVX rats (Wu et al., 2015).

We conducted an analysis of these formulas and identified *Lycium chinense Miller.*, *Epimedium brevicornu Maxim.*, and *Angelica sinensis (Oliv.) Diels.* as the most abundant components. Notably, the whole extract of *Sida cordifolia L*. and its most potent aqueous fraction were shown to significantly upregulate neuropeptides, including CGRP and SP, in nerve-injured rats exhibiting pain-like behavior (Tiwari and Hemalatha, 2024). Additionally, the combination of ferulic acid was found to reduce neurological deficits, decrease infarct volume, and inhibit the expression of IL-1β and NPY in a transient middle cerebral artery occlusion rat model (Ge et al., 2015). Furthermore, *Epimedium brevicornu Maxim.* and its active component, icariin, were observed to downregulate the expression of key proteins such as NPY, NPY1R, SP R, and 5-HT1B R, while significantly reducing VIP levels in a KOA rat model (Li et al., 2021b). Collectively, these findings provide strong evidence that these formulas play a role in the regulation of neuropeptides.

### 3.2 Single traditional Chinese medicine

The research on TCM and its effects on neuropeptides remains quite limited. *Plastrum testudinis* has been found to promote bone formation in OVX rats by reducing the expression of NPY and its receptor NPY1R in both brain and hypothalamic tissues, along with lowering NPY protein levels in femoral tissue (Li et al., 2022b). Additionally, *Epimedium brevicornu Maxim.* has been found to increase the expression of NPY, CGRP, VIP, and VIP2R in bone tissue, while reducing the mRNA expression of NPY1R, NPY2R, and TACR1. It also elevates protein levels of CRLR, VIP, VIP2R, and SP in bone tissue while decreasing NPY1R and TACR1 protein levels (Liu et al., 2018a).

### 3.3 Bioactive compound derived from Chinese medicine

Recent studies highlight the potential of bioactive compounds in modulating neuropeptide levels to regulate bone metabolism and improve bone health in OVX rats. Loganin, a bioactive compound derived from *Strychnos nux-vomica L.*, has been shown to significantly elevate serum NPY levels in OVX rats, thereby contributing to the regulation of bone metabolism and promoting a balance in bone remodeling (Xie et al., 2024). Similarly, Eleutheroside E, extracted from *Acanthopanax senticosus*, exhibits a dual role in enhancing bone health. It significantly increases serum concentrations of both NPY and procollagen type 1 N-terminal propeptide in OVX rats, indirectly influencing bone metabolism and resulting in improved bone mass (Zhou et al., 2023). Additionally, Eleutheroside E has also been found to elevate serum VIP levels in OVX rats, further regulating bone metabolism and promoting bone mass accrual (Zhou et al., 2023).

## 4 Conclusion

This review explores the roles of the neuropeptides NPY, VIP, CGRP, and SP in bone metabolism, as well as the TCM that can regulate these neuropeptides. However, current research is still limited. For one thing, the interactions among different neuropeptides and their specific regulatory mechanisms in bone metabolism have not been fully elucidated. For another, despite the potential demonstrated by TCM in regulating neuropeptides, its specific mechanisms and active ingredients still need thorough exploration. We thought that future research should focus on the following strategies. Firstly, the application of single-cell spatial transcriptomics is recommended to comprehensively map the heterogeneity of neuropeptide receptors across various bone cell subtypes. This approach will facilitate a detailed understanding of the molecular mechanisms underlying bone-nerve interactions at the cellular level. Secondly, the development of biomaterial-assisted delivery systems, such as mesoporous silica nanoparticles, should be pursued to achieve site-specific modulation of neuropeptides. This strategy has the potential to effectively overcome off-target effects, thereby enhancing the precision and efficacy of therapeutic interventions. Thirdly, the establishment of TCM component libraries paired with neuropeptide receptor CRISPR screening platforms is proposed to identify synergistic phytochemical combinations. This integrative approach will leverage the rich pharmacological diversity of TCM to identify novel therapeutic agents that can modulate bone-nerve crosstalk.

Our findings advocate for a paradigm shift from the traditional single-target inhibition approach to the restoration of multi-neuropeptide equilibrium. This shift positions TCM-derived formulations as precision modulators of bone-nerve interactions, offering a novel and potentially more effective therapeutic strategy. By bridging the current gap between mechanistic understanding and clinical application, this approach holds significant promise for improving the management of bone-related disorders, particularly in elderly patients with comorbid metabolic and neurological conditions.
